# Supplementary figures and images for: How synaptic strength, short-term plasticity, and input synchrony contribute to neuronal spike output
Source: PLoS Comput Biol. 2023 Apr 17;19(4):e1011046. doi: 10.1371/journal.pcbi.1011046 (PMC10153727; doi:10.1371/journal.pcbi.1011046)

**A**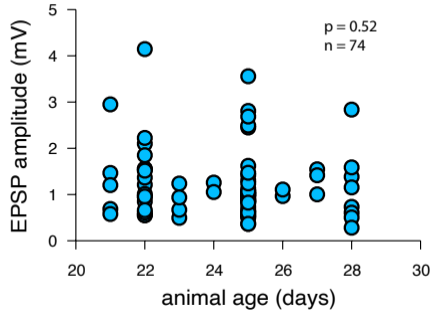**B**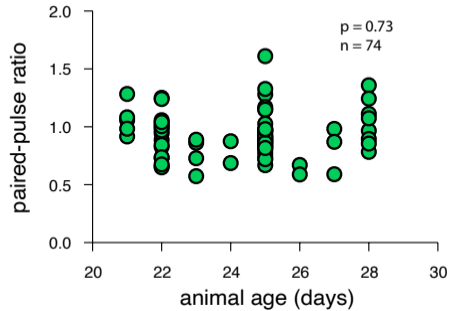

Supplement: S1 Fig — A EPSP amplitudes as a function of the age of the animals at the time of the experiment. B 20 ms paired-pulse ratios as a function of the age of the animals at the time of the experiment. Pearson correlation coefficients are indicated. (PDF) [file pcbi.1011046.s001.pdf]

**A**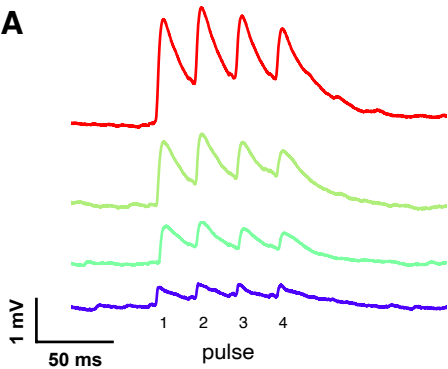**B**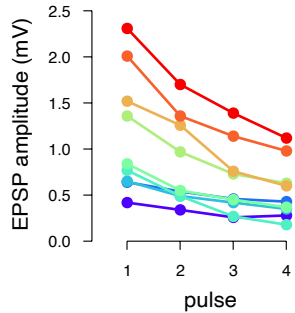

Supplement: S2 Fig — A Example traces of postsynaptic responses to trains of four action potentials fired in the presynaptic neuron at inter-spike intervals of 20 ms (i.e., 50 Hz; stimulation pulses indicated). Traces are averages and sorted and color-coded by decreasing EPSP amplitude (i.e., response to 1st pulse) and correspond to same colors in B. B All recorded synapses (n = 9) continue to depress during ongoing stimulation during trains of four action potentials fired in the presynaptic neuron. Data are sorted and color-coded by decreasing EPSP amplitude. (PDF) [file pcbi.1011046.s002.pdf]

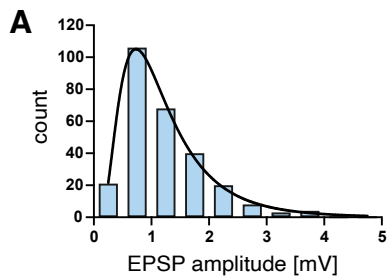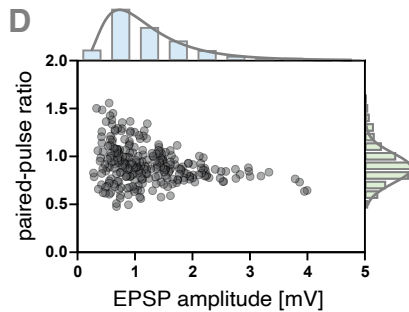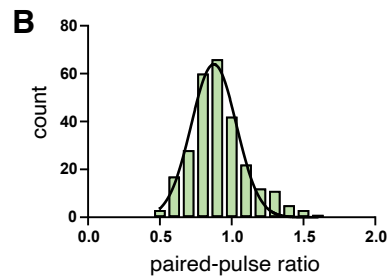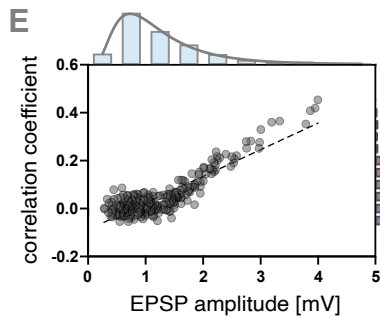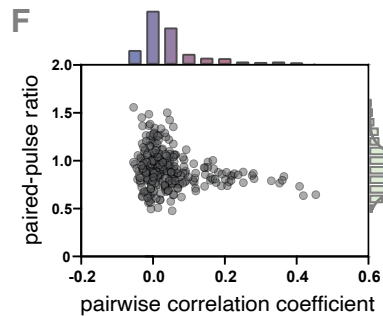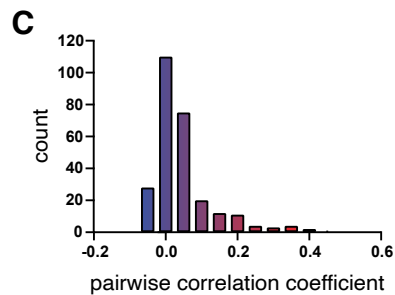

Supplement: S3 Fig — The relationships between parameter distributions reflects our in vitro data and in vivo data adopted from [6]. A EPSP distribution for 270 inputs generated from our in vitro recordings. B 20 ms paired-pulse ratio distribution for 270 inputs generated from our in vitro recordings. C Pairwise correlation coefficients for 270 inputs generated from in vivo data adopted from [6]. D Scatter plot of relationship between EPSP amplitudes and 20 ms paired-pulse ratios for the 270 inputs. E Scatter plot of relationship between EPSP amplitudes and pairwise correlation coefficients for the 270 inputs. F Scatter plot of relationship between 20 ms paired-pulse ratios and pairwise correlation coefficients for the 270 inputs. (PDF) [file pcbi.1011046.s003.pdf]

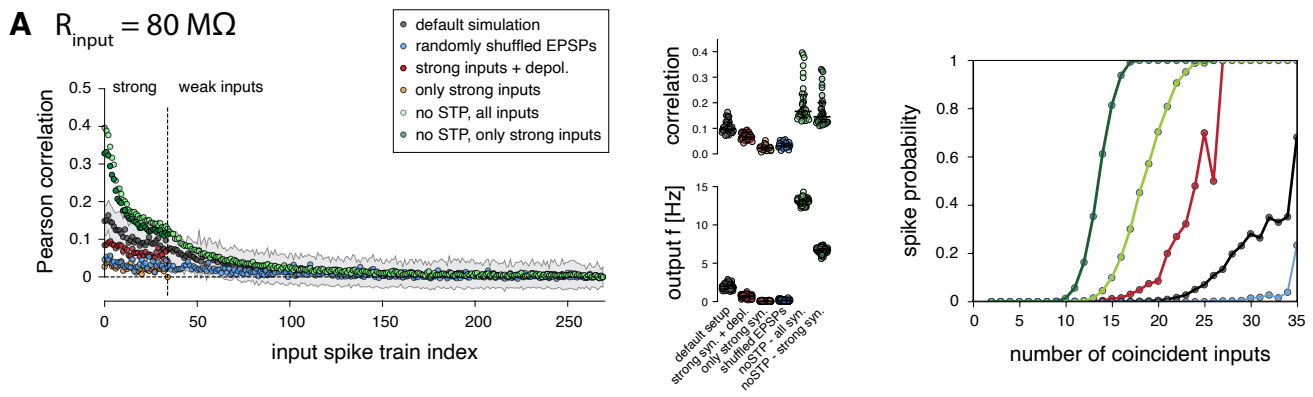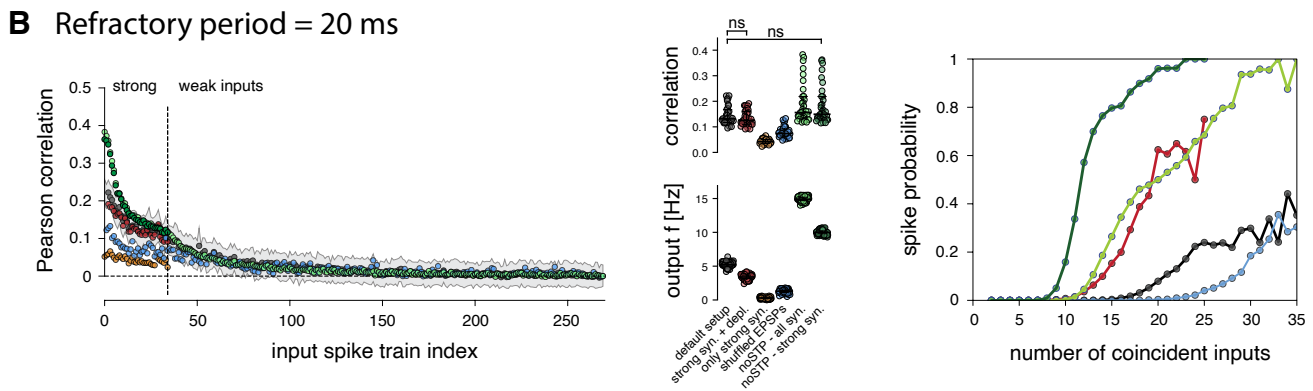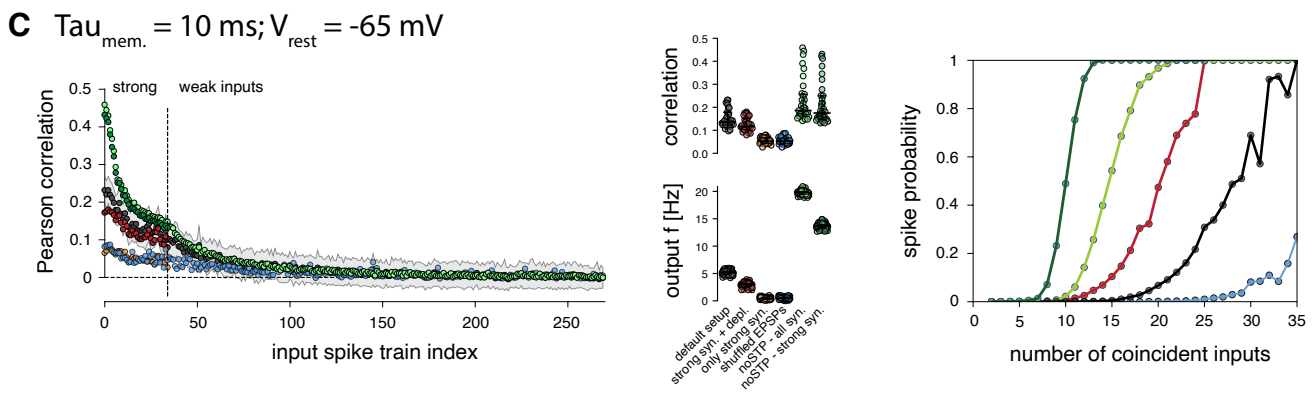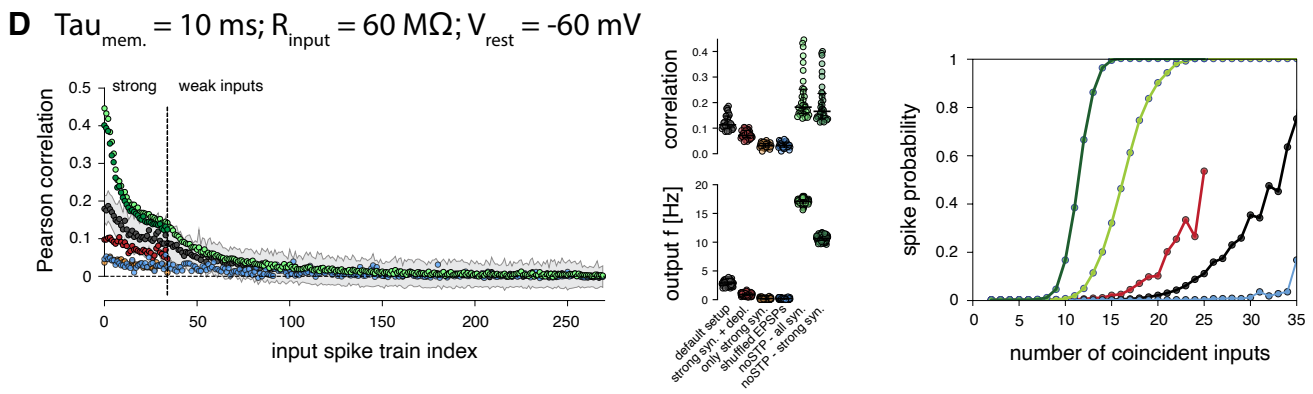

Supplement: S4 Fig — We changed individual (A, B), or combinations of biophysical parameters (C, D) of our leaky integrate-and-fire model and repeated all analyses shown in the Results section. Panel layout corresponds to panels in the bottom rows of Figs 5–7. In simulations shown in C, D, Vrest was depolarized to lift the output firing rate of the model cell > 2 Hz in the default setup, such that the correlation and gain analyses could be run. All pairwise comparisons between the experimental perturbations of the model and the default setup shown in the center panels (top and bottom) were statistically significant (p < 0.05, non-parametric Kolmogorov-Smirnov test) unless otherwise indicated. Shaded standard deviation bands were omitted for clarity, except for default simulation results in left panels. A Results for model with Rinput of 80 MΩ (instead of 100 MΩ), in agreement with recordings of barrel cortex L2/3 neurons in vivo [80]; all other parameters were left unchanged; note drop in output firing rate of model neuron (center-bottom panel). B Results for model with an ‘un-physiologically’ long refractory period of 20 ms following each spike event (instead of 10 ms). C Results for model with τm of 10 ms (instead of 20 ms), in agreement with recordings of barrel cortex L2/3 neurons in vivo [80]; all other parameters were left unchanged; Vrest was depolarized by 5 mV [9] to allow for sufficiently high output firing rate. D Results for an ‘in vivo-like’ model with Rinput of 60 MΩ (instead of 100 MΩ), τm of 10 ms (instead of 20 ms), and Vrest of -60mV, in agreement with recordings of barrel cortex L2/3 neurons in vivo [9,13,80]. (PDF) [file pcbi.1011046.s004.pdf]
