# Supplementary material for: How synaptic strength, short-term plasticity, and input synchrony contribute to neuronal spike output
Source: PLoS Comput Biol. 2023 Apr 17;19(4):e1011046. doi: 10.1371/journal.pcbi.1011046 (PMC10153727; doi:10.1371/journal.pcbi.1011046)
Supplement: Attachment — Submitted filename: Rebuttal.pdf [file pcbi.1011046.s012.pdf]

Department for Molecular and Cellular Biology
Harvard University
16 Divinity Ave
02138 Cambridge, MA
USA

Cambridge, USA, January 15th, 2023

Resubmission of revised manuscript PCOMPBIOL-D-22-01408

Dear Drs. Gutkin and Marinazzo,
Dear Referees,

Thank you for considering our manuscript *"How synaptic strength, short-term plasticity, and input synchrony contribute to neuronal spike output"* for publication in PLoS Computational Biology.

We have now addressed all comments and concerns raised by the referees and append a revised version of our manuscript, including our detailed responses to the referees' points, which are attached below.

We would like to sincerely thank the referees for their time and their excellent and detailed comments, which were invaluable for improving our manuscript.

We would like to briefly highlight two major improvements we have made to the manuscript:
(1) To address the concerns of Referee 1 regarding the use of minimal stimulation, we have included a dataset of synaptic measurements obtained with paired recordings of pyramidal neurons in barrel cortex L2/3. We found that both methods (paired recordings and minimal stimulation) produced the same EPSP amplitude and paired-pulse ratio distributions.
(2) Following the advice of Referee 2, we have simplified our modeling approach and are now using a leaky integrate-and-fire model. The Referee predicted correctly that this simpler model already reproduces all observed effects, and we agree that it is mathematically more tractable. Furthermore, we have included the parameter-robustness analysis, which both referees suggested, for this new model.

Yours sincerely,

Gregor Schuhknecht

Responses to Referees' Comments

Please find attached our detailed point-by-point responses to all comments made by the referees. For the convenience of the referees, the corresponding text changes in the manuscript are indicated by blue font color.

Reviewer #1: NOTE: We reviewed this manuscript for eLife and then for JNeurosci. Although our feedback changed between the eLife and the JNeurosci manuscripts, it appears that the present PLoS CB manuscript is virtually identical to the JNeurosci manuscript: figures, line numbers, etc appear to be exactly the same. We are therefore providing essentially the same feedback here as we did for the JNeurosci manuscript.

Please accept our apologies in advance if we forgot to remove points that were already addressed, in which case we would ask for a clarification as to how the point was addressed. We have seen so many versions of this manuscript now that it is easy to get confused.

Using a combination of acute-slice patch-clamp recordings, extracellular stimulation, and computer modelling, Guilabert et al explore how a neuron's spiking output is determined by synaptic strength, short-term plasticity, and spiking structure of its inputs. In contrast to the commonly held view that synaptic strength determines spiking output, the authors argue for a more nuanced view. In particular, the authors argue that they find that "the ability of strong inputs to evoke spiking critically depended on their high temporal synchrony and high firing rates observed in vivo and on synaptic background activity," meaning synaptic strength is just one factor among many that in practice that determine a neuron's spiking output.

This paper is well written and well argued. The figures were visually pleasing and straightforward to interpret. This made for a very pleasant and enjoyable read. The computational modeling revealed interesting findings that contribute to our understanding of how the integration of synaptic inputs contributes to cortical neuron output.

However, some claims seem a bit excessive, several of the modelling outcomes are presented as findings or evidence of realism when they seem to be a consequence of the parameter settings or alternatively not representative comparisons, the use of extracellular stimulation comes with limitations, and it is unclear why fast-spiking cells were included. Finally, the novelty is unclear, although this might be a matter of providing clarification. The study adds nuance to our thinking about what determines spiking output, even if we presumably still think synaptic weight mostly determines spiking output.

This manuscript should be highly suitable for publication in PLoS CB once these points have been addressed in full, so if the authors could address our concerns, we would strongly support publication.

MAJOR POINTS:

1. The authors make some strong claims from their data -- this could be clarified and/or toned down. For example, on p7, the authors say "these are important experimental results that contradict the theory-inspired hypothesis that synaptic inputs onto single cortical neurons may be statistically correlated", but it only seems to me that their data is "not consistent with" rather than "contradict", because I think of many caveats associated with the authors experiments (Major Points 2 and 3) and analysis (Major Point 5) that could also explain this mismatch. In general, it would seem appropriate of the authors to clearly outline the shortcomings and pitfalls of their own data and analysis.
We have toned down many of our statements across the manuscript and are now highlighting the caveats of our experimental and modeling approach in more detail. See for example line 129, line 255 (the example given by the Referee here) and lines 261f, 371f, 558f, 575f.

2. Extracellular stimulation is possibly an outdated method for studying monosynaptic connections, which leads to alternative interpretations of the present study. There are several issues associated with this methodology that the authors never control for or otherwise address. This should at least be discussed.
We agree that minimal stimulation is less well-controlled compared to e.g. paired recordings. It is important to stress, however, that we needed to use minimal stimulation because we wanted to record many different synaptic inputs to the same postsynaptic neuron, which is technically very challenging to achieve with paired recordings (e.g., line 124f).

We are addressing the concern of the Referee with a two-pronged approach: (1) we are explicitly addressing the shortcomings of minimal stimulation in the Results and Discussion (lines 261f, 575f) and (2) we are using paired recordings of L2/3 pyramidal cells as a control, which produced essentially the same results (Fig. 3 and Results, line 260f). This does not show that minimal stimulation in L2/3 produces completely unbiased results, but at least it suggests to us that the many caveats associated with the method did not contaminate our dataset in a significant way (Discussion, line 555f).

2a. How do authors know that inputs are independent? Then mention that stimulation locations are >50µm apart, but axonal arbours are considerably larger than this. In the older plasticity literature that relied on dual electrodes to show input specificity, independence of different inputs was verified with two electrodes activated in quick succession to show absence of short-term plasticity. Did the authors do any verification of that kind, or do we just have to take their word for it, that 50-µm spacing provides independence of inputs?
We cannot know for certain that all inputs are truly independent from each other, which we now acknowledge in lines 266 and 559. However, if we would have stimulated the same axon multiple times and recorded synapses repeatedly, then one would expect that we should have observed clustering of EPSP amplitudes and paired-pulse ratios on the same postsynaptic neurons (Fig. 2). The fact that we never observed such clustering suggests that most synaptic inputs we recorded were likely independent of each other (line 564f).

2b. PPR should be affected by extrac stim under some circumstances. With minimal stimulation, afferents can easily drop out, but when they do, they can differentially drop out on the first or the second pulse of a paired-pulse stimulation, which will apparently affect the paired-pulse ratio. Given that they authors report a correlation between the EPSP amplitude and the PPR, they need to account for the possibility that this is a consequence of using extrac stimulation rather than paired recordings.
To address this concern, we analyzed the data obtained with paired recordings and found the same relationship between EPSP amplitude and PPR (Fig. 3), suggesting that this correlation is not an artefact of using minimal stimulation.

2c. Was excitation ever contaminated by inhibition? If no, how do the authors know that excitation was not contaminated by inhibition? Did they wash in GABA blockers to show that inhibition was never recruited, or only recruited so often? Did the authors carry out any other control?
We have specified that we did not include GABA blockers because our new paired recording data and previous minimal stimulation literature suggest that inhibition likely did not contaminate our dataset in a significant way (lines 706f, 716f).

2d. Anybody who has ever used extrac stim knows that the CV of responses decreases with increasing stim strength, as afferents are averaged and noise thereby reduced. Do the authors argue that these are unitary inputs, or a mix? If the authors wish to argue that minimal stimulation only recruits unitary inputs (like with a paired recording), then they need to report controls to that effect. If the authors do not wish to argue that recordings are unitary, then they are dealing with a mixed data set, where some recordings may be unitary, but some are not, which would impact the heterogeneity of the data set. This could for example help to explain why the PPR of large (>2mV) inputs show a smaller PPR variance, since they are the consequence of multiple inputs being averaged, leading to smaller CV. Larger PPR spread of small inputs could be due to more noise -- since the PPR is a ratio, it is sensitive to the pulse-1 amplitude in the denominator.
We still cannot completely rule out that some of the synaptic connections we activated with minimal stimulation could indeed consist of multiple different axons, which we have acknowledged in line 261f. However, in adding the paired-recordings dataset, we hope we are providing the control experiment the Referee mentioned. As EPSP amplitudes and paired-pulse ratios did not differ between the minimal stimulation and paired recordings datasets, our interpretation is that the paired recordings show that synapses recorded with minimal stimulation were in general unitary. Therefore, we would like to argue that we recorded putatively single axons of passage with minimal stimulation (line 566f).
As the Referee suggests, we have indeed found a negative correlation between PPR variance and EPSP amplitude in our minimal stimulation data (see figure below; left). However, the same negative correlation was also present in our paired recordings (see figure below; right), which suggests that it is likely not an artifact due to minimal stimulation, but instead has to do with different quantal transmission properties of strong versus weak synapses.

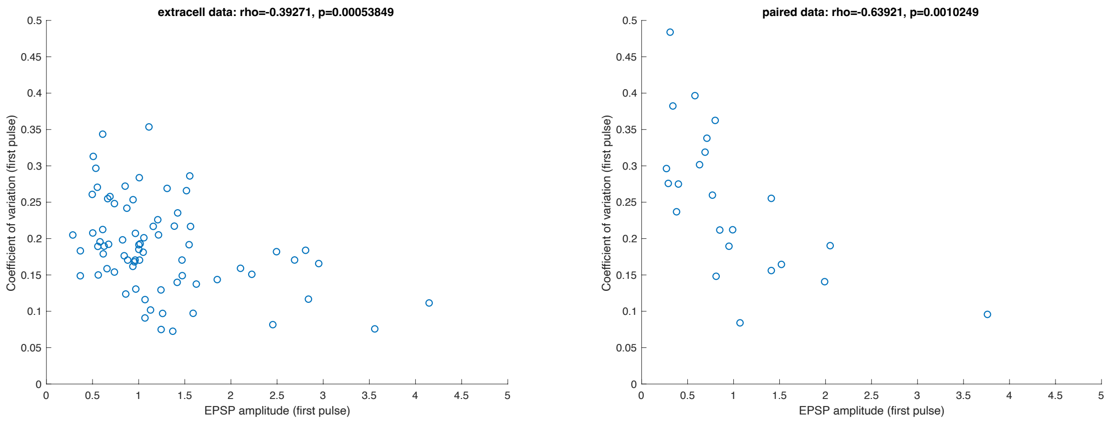

2e. How do the authors really know the identity of the presynaptic cell? With paired recordings, we'd ask for morphometry, spike pattern, layer localization, and/or genetic markers of the presynaptic cell. Here we just have to believe that the stim electrode location tells all we need to know. Maybe the heterogeneity of inputs is a consequence of activating different kinds of inputs?

We agree that it is clearly a caveat of minimal stimulation that we cannot know the identity of the presynaptic axon, which we have explicitly acknowledged in line 261f. Nonetheless, our paired recording experiments of identified pyramidal neurons in barrel cortex L2/3 (Fig. 3) suggest that the synaptic population sampled with minimal stimulation (Fig. 1) is comparable to synaptic connections between L2/3 neurons (lines 267f, 555f).

3. Paired pulse ratio is not the same thing as the full short-term dynamics of a synapse. It is both possible and quite common for a synapse to exhibit short-term facilitation in a paired-pulse sense while still showing short-term depression overall for the remainder of a high-frequency burst. In fact, already the Tsodyks-Markram model famously shows how assessing short-term plasticity merely from the first two pulses can be misleading. The authors need to address this measurement problem.

Indeed, the thalamocortical projection to L4 of barrel cortex has been shown to display 'diverse' short-term plasticity: some connections that are depressing from rest will show facilitation during ongoing activation (Díaz-Quesada et al., 2014). To test if this could happen in the recurrent L2/3 circuitry as well, we stimulated the presynaptic neuron with trains of four action potentials in our paired recording experiments. We never observed such diverse short-term plasticity; instead, all cells that showed depression for the first two pulses from rest continued to depress during ongoing stimulation (line 298f, Supplementary Fig. 2).

4. It is unclear why fast-spiking cells were included in this study. The authors report that excitatory synaptic connections formed with regular-spiking L2/3 neurons do not exhibit a systematic clustering of EPSP amplitude and short-term plasticity. Did they find the same results for the excitatory connections onto fast-spiking cells? Those results should be reported too. The authors show data for EPSPs onto fast-spiking neurons in Fig 1, but then they never mention fast-spiking neurons again and do not provide any explanation as to why they did not pursue any further investigation into the integration of inputs onto fast-spiking neurons. Are those cells ever mixed up with the pyramidal cell recordings? That would seem inappropriate. I am thinking that it might be a good idea to simply remove the fast-spiking cells from this study.

As both referees suggested, we have removed the fast-spiking cells from the manuscript. We agree that these data were likely confusing rather than helpful or interesting.

5. The statistical treatment to show that there is no clustering is at times unclear.
- First of all, Fig 2C shows how the cell 1 data is included in the population data for the comparison between cell 1 and the population, which clearly violates the requirement that the data sets be independent. The same holds true for the cell 2 - 8 comparisons. In the Methods on p22, HOWEVER, something else is stated: "To obtain an unbiased population distribution for a given experiment, we excluded all afferent synaptic connections formed with the postsynaptic neuron in that experiment, but otherwise included all other connections recorded in regular spiking neurons." Please clarify this in Fig 2. Maybe we are just misunderstanding what was done and it is all good?

Thank you for pointing this out. We have clarified this in the figure legend of Fig. 2. As explained in the Methods, we always removed the synaptic properties recorded in a particular cell from the population distribution.

- Second, the power analysis relies on two approaches, but neither of them require information about the

overall number of inputs that exist onto a L2 PC. Please justify this approach. Cerebellar granule cells, for example, famously have only a handful of inputs. If the authors had been recording from granule cells, they would thus be able to sample a much, much larger fraction of the overall input 'connectome' onto those cells than they now can with L2 PCs. According to Braitenberg and Schuz's many papers, your typical pyramidal cell should have something like 5000 to 10000 inputs. Presumably the power analysis of the clustering must depend on how well the overall input space is sampled, yet this parameter never enters the picture. Please clarify.

As the Referee suggests, each cell receives thousands of synaptic inputs, but we can record experimentally only from a handful of them, i.e., 5-8 synapses. We assumed, for the sake of simplicity, that this process is captured sufficiently accurately by 'sampling with replacement' from an underlying probability distribution (that describes either EPSP amplitude or PPR). In other words, if the number of recorded synapses is much smaller than the total number of synapses, the total number of synapses formed with these cells should be negligible. Then, the distribution of synaptic parameters can be approximated with a probability distribution instead of a discrete numerical distribution with a finite number of entries. We agree with the Referee that, if we had recorded from cell types with few inputs, then the statistical test should indeed depend much more strongly on the total number of synaptic inputs a cell receives.

- Third, PPR depends on age over P14-P30 (e.g. Reyes & Sakmann 1999), and the authors explore P22-P29. Does PPR correlate with age in the authors' data set? Could this affect the clustering test?

We checked this and found no correlations between age and EPSP amplitude or age and PPR (line 171f; Supplementary Fig. 1). This could be because our age range is much narrower than the one used by Reyes and Sakmann.

- Fourth, repeated comparisons are done in Fig 2, so how are post-hoc corrections for multiple comparisons done? If you keep testing, eventually a comparison will come up significant. Page 22 says "Because our dataset contained 8 experiments for which at least 5 afferent connections...", which seems to claim that this took care of this issue, but does it really? Please clarify.

We did indeed not correct for multiple comparisons in Fig. 2 A-B. (None of the eight comparisons were statistically significant and they would continue to be not significant if we corrected for multiple testing.) Importantly, however, the take-home-message of the figure is the analysis shown in panel C, which does in fact account for multiple testing by asking: "Which effect size should have produced a statistically significant difference at the 95% certainty level in at least one of the eight experiments?". This is explained in line 208f. We think that the detectable effect size across the entire experimental series is the most meaningful metric to report.

6. Several of the modelling outcomes are presented as findings or evidence of realism when they are simply a consequence of the parameter settings or alternatively not representative comparisons.

We have changed our phrasing across the modeling section of the manuscript, please see our comments below for details. Please also note that we have replaced our 'ball-and-stick' neuron model with a leaky integrate-and-fire model, following the suggestion by Referee 2. The leaky integrate-and-fire model produced all the effects we reported in the previous version of the manuscript, and we agree with Referee 2 that such a simpler model is mathematically more tractable and should be a better fit for our study.

6a. On p8, the authors state "The mapping between EPSP amplitude and short-term plasticity across the model inputs (Fig. 4 A, B) followed the same relationship as observed in vitro". It would be very strange if this was not the case, since this is how and why the model was constructed. Or am I missing something here?

The Referee is correct, and we realize that this phrasing can be very confusing and have removed it. This statement was meant to provide a sanity check that the model was set up as intended. We also moved the associated figure to the supplementary materials.

6b. On p10, the authors say "Critically, without further tuning, the model neuron reproduced key properties of rodent L2/3 pyramidal neurons in vivo", but R_input, tau_membrane etc span huge ranges in the acute slice, it is one of those famous long-tailed distributions, so that the authors happen to get a good match is just luck -- it cannot possibly mean anything. To convince themselves that this is pure luck, the authors should carry out a robustness analysis with respect to key parameters of the model (axial resistance, R_i, gleak at dendrite and soma, etc) -- this will show how much the firing rate can actually vary. The authors can for example start with picking an axial resistance of 70 Ohm-cm instead of 100 Ohm-cm (p23), or tweak R_i by 5% away from 1 Ohm-cm, or why not nudge the gleak of the soma something other than the arbitrarily precise selection of 0.0379 mS/cm2. Besides, many cited parameter values come from in-vitro measurements, but e.g. R_input and tau_m are famously totally different in vivo, due to background activity, so the fact that modelling and in-vivo frequencies magically match is actually evidence that they do not match.

Of course, the Referee is entirely correct that the output of the model is determined by how we chose its parameters. We have specifically pointed this out in the manuscript (e.g. line 371f).

We have removed this phrasing on p10 and we are specifically stating now (line 382f) that we set V_m (to -70mV) such that the output spike rate of the model becomes ~ 5 Hz (as observed in vivo).
We have also conducted the power analysis that was suggested by both referees (Supplementary Fig. 4) to investigate how exactly parameter tuning affects our results. To that end, we adopted the strategy suggested by Referee 1 here. The model was initially set up and run using R_{in} and τ_m that we measured in vitro (lines 317f, 381f). We then observed how changing R_{in} or τ_m to values reported for L2/3 neurons in vivo changed the results. We also varied the refractory period to an artificially long duration to show this had no major implications on the results. Finally, we simulated the model in a 'realistic' in vivo setup, in which both R_{in} and τ_m were set to very low values and V_{rest} was more depolarized. In summary, we found that while the absolute firing rate of the model depended on the specific parameter settings, the qualitative results of our correlation and gain analyses were largely unaffected by parameter tuning – as long as the model was allowed to spike > 2 Hz in its default setup (which we achieved by depolarizing V_{rest} accordingly).

6c. On p11, the authors say "The average membrane voltage (Vm) of the model neuron was -65.93 mV ± 7.82 mV (Fig. 5 B, D), comparable to in vivo whole-cell recordings in mouse L2", but in-vivo recordings are done during synaptic bombardment, under unclear up/down state, and anaesthesia. While it is good to have realism, I am not sure how much we can learn from this.
We have specified now that the model is also under synaptic bombardment of the background inputs while we measured its V_m (line 385f). We have left this information in the manuscript because we think it could convince readers that our specific choice of parameters results in a model that recapitulates certain behaviors of L2/3 neurons in vivo.

6d. On p11, the authors say that when they removed the weak inputs, "The output firing rate of the model neuron dropped to 1.26 ± 0.34 Hz (Fig. 5 F) and its average Vm was hyperpolarized to -68.39mV ± 4.57mV." The authors now need to compensate for this change in V_m and R_input due to removing the weak inputs by adjusting the model accordingly, otherwise the findings are trivial -- there was just an indirect change in model parameters. Correct me if I am wrong, but I cannot see that the authors carry out such a compensation. The cited Destexhe papers have a method for such a compensatory change, for example. But it would seem simple to depolarize the cell accordingly to compensate for loss of average conductance drive, to enable comparison. Otherwise it would seem odd to claim that "Notably, synaptic strength alone did not determine which presynaptic cells could evoke spikes (Scholl et al., 2020)" (page 17).
We would like to thank the Referee for suggesting this control because we think it provided some interesting results. To clarify, we are indeed arguing that the computational effect of the weak inputs relies partly on depolarizing the membrane and bringing the cell closer to threshold, which may be trivial. Therefore, we have kept the modelling setup in which we completely remove weak inputs without compensation. In a new modeling experiment, we have now included the compensation suggested by the Referee (line 428f, Fig 5): we removed the weak inputs and depolarized the membrane to the median resting potential that we observed when only the weak inputs were active. The results show that the spike rate of the model drops, while the basic correlation structure is preserved. This experiment is interesting, because it allows us to distinguish between two separate mechanisms by which the weak inputs amplify the strong inputs: (1) by simply depolarizing the membrane and (2) by stochastic membrane fluctuations (that are removed in the setup suggested by the Referee).

6e. P17, "such that it was not necessary to include inhibitory synapses in the simulation to balance excitation." and then "Reassuringly, without further parameter tuning, the model exhibited key computational properties of cortical neurons that have been characterized in experiments and simulations before: the model cell produced sparse firing at around 5 Hz, which is in excellent agreement with the average spike rate reported for mouse barrel cortex L2/3" Honestly, doesn't it come across as super weird that model firing rates match in-vivo firing rates if the model doesn't even have inhibition? This line of reasoning seems odd to me.
We agree with the Referee that these statements were misleading as they suggested a level of realism that the model doesn't aim to provide. We have therefore removed them from the manuscript. We have also specified that the model aims to investigate the excitatory component of the L2/3 circuitry (e.g. line 315).

6f. P27, "Reassuringly, we found that the resulting somatic EPSP amplitude distribution and paired-pulse ratio distribution exactly matched the target distributions we had generated". I don't get it. Why wouldn't it match? That whole paragraph starting with "By deriving paired-pulse ratios..." is not reassuring to me, it confuses me.
See our reply to comment 6a of Referee 1: also this sentence was intended as a 'sanity check' that the model was set up as desired. We agree that it could be confusing to readers, and we have therefore removed it.

7. Calcium has a major impact on short-term plasticity. The authors use 2.5 mM, but make comparisons with papers that use other concentrations without mentioning this (e.g. Lefort and Petersen use 2 mM, see p16). This caveat should be made clear.
Thank you for raising this important point, we are discussion this possibility now (line 575f). The Ca concentration could indeed influence our results, although we think this is probably unlikely to have a strong effect, given that Reyes and

Sakmann (1999) and Feldmeyer et al. (2006) also find average depression, but are using the lower Ca concentration of 2 mM, like Lefort and Petersen, who report facilitation on average.

8. The novelty should be brought out better. The study adds nuance to our thinking about what determines spiking output, but it does not really change the way we think. Presumably, we still think synaptic weight mostly determines spiking output, no? Please clarify.
Thank you for pointing out this important issue. We have clarified the novelty of our study across the Abstract (lines 9 & 15f), Introduction (line 101f) and Discussion (lines 537, 545, 678f).
Firstly, we have now mapped synaptic strength, short-term plasticity, and how they relate in detail in barrel cortex L2/3 with two different experimental techniques, and we hope these datasets will be of interest and value to the neuroscience community (line 537f).
Secondly, we believe that our finding that there is no pronounced clustering of EPSP amplitude and/or PPR on different pyramidal cells is interesting for theoreticians, who have proposed this as a potential mechanism for implementing filtering functions or to explain lognormal firing rates in the cortex. To date, this has not been tested experimentally. The fact that our results are not consistent with these theories could thus provide important experimental constraints on future models (lines 9 & 545f).
Finally, because the strong synapses are all depressing, this should limit their 'power' during continuous activation. Our modeling results suggest that strong synaptic weight alone, when considered in isolation, plays a weaker role in driving correlated firing in the postsynaptic neuron compared to the combination of high correlation and high firing rates in the L2/3 circuitry. Interestingly, the same has been found for the thalamocortical connection: thalamocortical synapses are stronger in vitro compared to corticocortical synapses (Stratford et al., 1996). However, in vivo, they are continuously pre-depressed due to high thalamic firing rates and spontaneous activity (Boudreau and Ferster, 2005), such that they have low efficacy and require the high temporal correlation and high firing rates of thalamocortical neurons that 'do the heavy lifting' to drive L4 (Bruno and Sakmann, 2006; Banitt et al. 2007).
Importantly, however, we note that by pairing the correlated, 'driving' inputs with the largest EPSP amplitudes in our simulation (despite short-term depression), the information transfer of the driving inputs and the responsiveness of the postsynaptic neuron are markedly amplified (line 15f). Therefore, the Referee is correct that here our study adds nuance to our understanding (lines 18f, 678f); for example, by proposing that even under considerable depression, synaptic strength has an important amplification effect and could be a mechanism to sharpen the response properties of pyramidal neurons.

MINOR POINTS:

7. p4, how was the 10-90% rise time measured? From the average sweep, or from the individual sweeps and then averaged? If the former, this will due to temporal jitter tend to make the rise time longer. Please clarify.
The rise times were measured from individual sweeps and then averaged; this was clarified in line 770f.

8. p2, "interval of 20 ms", it seems more meaningful to report the frequency.
We are now reporting the respective frequency in line 169.

10. p4, "a scatter plot of the response amplitudes to the 2 stimulation pulse against the response amplitudes to the 1st stimulation pulse..." This is also true for individual sweeps (at least for paired recordings between pyramidal cells), because the readily-releasable pool depletes less if there is failure of release on the first pulse, so that the readily-releasable pool is closer to full for the second response, and vice versa. A corollary seems to be that lower PPR for large EPSPs may be a consequence of p_release being close to maxing out for strong synapses. By analyzing the individual sweeps, the authors may be able to show this: weak inputs may have a strong correlation between pulses 1 and 2, whereas strong inputs may not, due to maxing out p_release, thus leaving little or no space for pulses 1 and 2 to correlate.
We have done this analysis, but the results are inconclusive, and we therefore prefer to not include them in the manuscript. While there seems to be a trend that strong synapses are less correlated between pulses 1 and 2, as the Referee suggested, there is no significant correlation; and this is true for both our minimal stimulation experiments (left) and our paired-recording experiments (right):

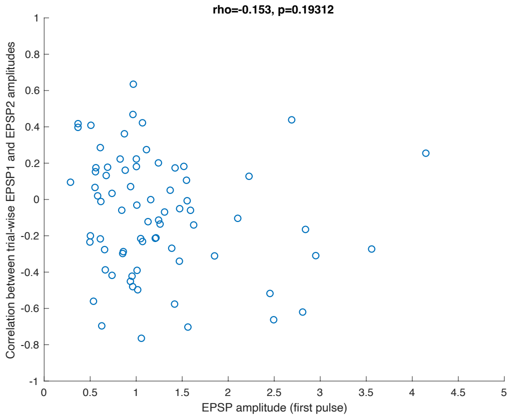

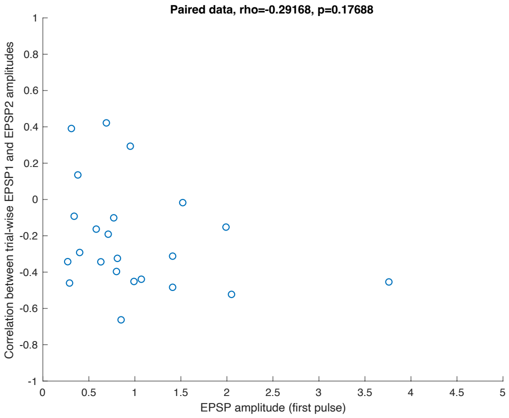

11. The authors are often using the word "significant", which is confusing. In science, findings are there if they are significant, and not there if they are not significant, so there is no need to say that e.g. (p4) "There was no significant correlation in our data set", it is enough to say "There was no correlation in our data set". The reason this is important is because sometimes significant just means "a lot", but on p5, with "connections above 2 mV had a significantly lower mean paired-pulse ratio", I can't tell if the authors mean "a lot lower" or "statistically significantly lower". Therefore, I recommend just staying away from the s-word, "significant", unless there is some sort of special reason for using it. To denote "a lot", the authors could say "considerably" or "markedly" etc.
We agree that this could easily be a reason for confusion and we have replaced all arbitrary uses of the s-word with synonymous expressions (e.g. line 64) or have removed the word altogether. However, in some instances, we believe it makes the text easier to follow when the word ‘significant’ is used and in those cases, we are using it strictly in the statistical sense, in accordance with the policies of most scientific journals (i.e., in the chapter on synaptic clustering, or the Methods section).

12. Fig. 1C, F: I would like to see the p-values for those correlations in the graph or in the figure caption.
The p-values for Fig 1 F are shown in the figure panel. We did not run a correlation analysis on the data shown in Fig. 1 C (the scatter plot of EPSP 2 versus EPSP 1), the data were simply plotted for visualization.

13. p5, "To investigate this further, " should probably start a new paragraph.
Done. (Line 184 and also line 238.)

14. I do not quite understand how these panels related to the figure caption just below. Could the authors explain in the caption what these results mean, please?
We are not sure which figure panels are referred to, but we are more than happy to clarify if the Referee could specify this.

15. Query, p13-14, Was short-term plasticity initial conditions run until it was relaxed, or did it start naively for each simulation run? Please clarify.
After each run of the simulation, short-term plasticity was reset and started ‘naively’. We clarified this in line 933f.

16. P16, unclear what "entire L2/3" means here.
We clarified this in line 580f; we meant the entire ‘extent’ or ‘thickness’ of L2/3. See also line 584.

17. P17, "the intriguing possibility that statistical biases of synaptic innervation may exist on the level of dendritic branches", please cite relevant papers. I think David DiGregorio had a paper back in 2012 showing that Purkinje Cells dendritic filtering affects PPR, I imagine similar papers must exist for neocortical pyramidal cells. Maybe try Stephen Williams and/or Greg Stuart.
We have cited three relevant papers here, including Abrahamsson et al. (2012), as suggested by the Referee.

18. p23, Where are the synapse located along this model dendrite? Does the synapse location affect the PPR (it would if the dendrite has e.g. I_h, like cortical pyramidal cells do)
We assume this concern has been resolved with the use of the integrate-and-fire model.

19. P18, paragraph starting “Our result that spiking in the model neuron was driven...”, How would the

computer model be different if based on Scholl et al 2020 findings? I mean, does the line of reasoning presented in this paragraph really explain the difference? I smell a non-sequitur here, but maybe I am missing something?
The modeling setup in which EPSPs are randomly shuffled across all spike trains was inspired by the results of Scholl et al. In this setup, the correlated, driving inputs have EPSPs of varying strength that are not systematically larger than those of the weak inputs. We have clarified this in the respective paragraph in the discussion (line 649f).
It is interesting that the correlated inputs still drive the output cell, and that they do so less strongly in our simulation. This suggests that other amplification mechanisms, for example the dendritic clustering of smaller synapses observed by Scholl et al., may play an important computational role in compensating for the 'lack' of synaptic strength (line 651f).

20. P20, please report the liquid junction potential, even if not corrected for.
Reported now in line 713.

21. P20, "If the membrane potential drifted during recordings, a holding current was injected to keep the membrane at its initial resting potential, which was rarely necessary." Please state how much. Please clarify: Was V_m allowed to drift outside -85 to -70 mV?
Vm was not allowed to drift outside that range (line 715f) and the holding current has been listed (line 714).

24. P21, vague minimal stim criteria, "in an all-or-none manner" and "which showed little or no variability in EPSP latency or shape". Please provide specific numerical values.
We have clarified our protocol for minimal stimulation (line 725f) and, for the reference of the readers, have cited several of the seminal hippocampus papers that relied on minimal stimulation to investigate the origin of long-term plasticity (on which we based our protocols). We found that in the minimal stimulation literature, it is not common to provide specific values here. We think this makes sense, because during the experiment, the choice of the exact stimulation current is not determined by a specific, pre-determined fraction of trials that lead to release (which would also depend on the release probability, and thus be different from synapse to synapse). Instead, these choices have to be made by the experimenter during each new recording on the basis of experience; therefore it may be misleading to report specific values. However, if the Referee disagrees, we could offer to measure e.g. the fraction of successful release events and variability in EPSP latency post-hoc in our dataset.
Moreover, we believe that the agreement of our minimal stimulation and paired recording data, and the lack of clustering in Fig. 2 suggest that our minimal stimulation criteria were chosen adequately such that mostly single axons were stimulated (see above).

26. P22, "to identify different axon fibers" and "Great care was taken not to record from the same" -- How do you know it is different, really? See Major Point 2.
As pointed out in line 263f, we cannot be sure that all inputs are truly independent from each other. Additionally, we specified in the Methods section that these steps were taken to minimize the chance of recording multiple times from the same axon, rather than 'ensuring' that we can actually record independent inputs (line 742f).

27. P23, "We convolved the template spike train with Gaussian envelopes..." Isn't this vernacular use of the word "convolve"? Two instances.
The mathematically correct term would be 'to convolute', we have changed 'convolve' to 'convolute' in lines 840, 851, and 981.

29. P24, a thought: Increasing the rate of a Poisson process also lowers its CV and would tend to artificially enhance cross-correlations. How was this accounted for?
We did a control experiment to verify that our correlation analysis indeed picked up correlation in spike timing, while the effect of elevated firing rates was negligible: we randomized the output spike train of the model neuron and ran the same correlation analysis as in the other modeling experiments. We found that the correlation coefficients of all inputs dropped to zero. If the higher spike rates of the stronger inputs would lead to an increase in correlation, then the stronger inputs should have displayed higher correlation coefficients compared to the weak inputs; see line 393f.

Reviewer #2: This paper combines experimental work on short term synaptic dynamics and single cell modelling to investigate synaptic integration in L2/3 cells.
After measuring synaptic dynamics, they use a 2 compartment model to measure synaptic integration. A very specific pattern of activity is used where strong synapses receive higher rate have less jitter and are more strongly correlated. (Little attention is given to higher order correlation in the inputs that will become dominant at some point).
Unsurprisingly in this setup, the many background inputs lift the membrane potential of the cell and boost the effect of the strong inputs.
The net effect of STP is weak. While the effect of the background inputs becomes weaker this likely depends on precise model parameters.

The paper is clear, but the conclusions are not particular surprising from experimental or computational point, novel, or solid. Namely, synchronous, high rate inputs are effective in driving the neuron, and a background input helps to lift it above threshold.
We have clarified the novelty of our study across the Abstract, Introduction, and Discussion.
Briefly, we think our study contributes to a more nuanced view of the interplay between synaptic strength, short-term plasticity, firing rates, and synchrony (lines 15 & 678f).
At the same time, we believe our finding that there is no pronounced clustering of EPSP amplitude and/or PPR on different pyramidal cells may be of interest to theoreticians, who have proposed this as a potential mechanism for implementing filtering functions or to explain lognormal firing rates in the cortex. These data could provide experimental constraints on future models (lines 9f, 101f, 545).
Finally, we have now mapped synaptic strength, short-term plasticity, and how they relate in detail in barrel cortex L2/3 with two different experimental techniques, and we hope these datasets will be of interest and value to the neuroscience community.

Because the biochemical milieu can be quite different in in vivo and in vitro (e.g. adenosine to name one), and hence dynamical synaptic properties can vary a lot, the extrapolation from in vivo to in vitro without further verification is rather risky.
We have conducted the robustness analysis that was suggested by both referees and have simulated our modeling results for different ranges of parameter settings. One of these tested models was tuned entirely after experimental measurements of the biophysical properties of L2/3 pyramidal cells in vivo (see below for more detail).

Major:

The emergence of a correlation only when RS and FS cells are pooled, but not in the individual FS or RS population by itself is troublesome (Fig 1F). It suggests that an confounding explanation is that FS cells have large, depressing synapses and RS cells small, less depressing synapses.
Can this be ruled out? If not, how would that affect the result?
Following the concerns and advice of both Referees, we have removed the fast-spiking cells from the manuscript.
Our interpretation is that the correlation between synaptic strength and paired-pulse ratio of pyramidal cell synapses in L2/3 is weak. We added a new analysis to Fig. 1 F that supports this: the correlation between average EPSP amplitudes and average PPRs does not reach significance; however, when we analyzed the individual sweeps, there is a significant correlation – likely because the dataset is much larger. Therefore, we believe that the relationship between synaptic strength and short-term plasticity in our data is best captured not by a correlation analysis, but instead by the statement that strong synapses are depressing, while weak synapses display the full range of short-term plasticity.
We have also added a novel dataset to the manuscript that consists of EPSP and PPR measurements obtained with paired recordings in the same tissue (Fig. 3). These data act as a control experiment for the minimal stimulation, which has several caveats (as pointed out by Referee 1), because they show that the minimal stimulation experiments provided similar results as paired recordings of synapses between L2/3 pyramidal cells: in both datasets, there is a correlation for synapses analyzed on the single-trial level, and strong synapses are depressing, while weak synapses show a full range of short-term plasticity (Fig.1 and Fig. 3).

It would be of interest to know whether a simple linear integration model would already predict already all the observed effects. I would assume so. Currently the model is too complicated to understand mathematically, but too simple to be a good model of a neuron.
It does! We have changed our modeling approach to using a classical integrate-and-fire model, as the Referee suggested. Indeed, all observed effects could be reproduced with this simpler model (Figs. 4-7). We agree that because the integrate-and-fire model has fewer parameters and because it is mathematically much simpler, it should be a better fit for our manuscript and will likely make our results more easily interpretable and easier to reproduce. We would like to thank the Referee for this very helpful suggestion.

Minor:

l135. I don't see how the question of correlation/mapping is dependent on the clause ("Given their...").

We have corrected this, now in line 174f.

I wonder why in the model Ri in the soma was set to 1 Ohm cm. It really should not matter given the large soma which should be close to electrotonically compact. (If it does there is also certainly something wrong). Also, if the membrane capacitance was enhanced to compensate for the presence of spines, why was the somatic conductance boosted even more?

I was also surprised by the choice of making all synapses equidistant. This will increase saturation and makes one wonder whether a spatially extended model was used at all.

These concerns have likely been resolved with the use of the integrate-and-fire model.

l778 The statement that no robustness analysis is needed, because synaptic strenghts were tuned to data is misleading and very wrong. While the indivual synaptic strengths might have matched, properties such as synaptic interaction and filtering will dependent on the precise parameter combination. If the statement were true there would be no need for detailed models at all.

Following the advice of both referees, we have now conducted a robustness analysis to test how different parameter combinations of the integrate-and-fire model influence the results (Line 371f, Supplementary figure 4).

To that end, we adopted the strategy suggested by Referee 1. The model was initially set up and run using R_{in} and τ_m that we measured in vitro (line 317f, 823f). We then observed how changing R_{in} or τ_m to values reported for L2/3 neurons in vivo changed the results. We also varied the refractory period to an artificially long duration to show this had no major implications on the results. Finally, we simulated the model in a 'realistic' in vivo setup, in which both R_{in} and τ_m were set to very low values and V_{rest} was more depolarized.

In summary, we found that while the absolute firing rate of the model depended on the specific parameter settings, the qualitative results of our correlation and gain analyses were largely unaffected by parameter tuning – as long as the model was allowed to spike > 2 Hz in its default setup (which we achieved by depolarizing V_{rest} accordingly).

l830 I presume the sd has also a factor 10^-6.

The standard deviation is indeed ± 0.192. We measured the jitter of datapoints around a fitted curve. Datapoints on different sides of the curve were given different signs. Therefore, the distribution of jitter is centered around zero, which we have now clarified to avoid confusion (line 879f).
